# A Cross-Sectional Evaluation of the Association between Orthodontic Treatment, Retention Modality and the Prevalence of Gingival Recession

**DOI:** 10.3290/j.ohpd.b5871487

**Published:** 2024-12-05

**Authors:** Panagiotis Theodorelos, Martina Ferrillo, Nikolaos Pandi, Dimitrios Kloukos, Padhraig S. Fleming, Christos Katsaros

**Affiliations:** a Panagiotis Theodorelos Periodontist, Department of Periodontology, 251 Hellenic Air Force & VA General Hospital, Athens, Greece. Data acquisition, recording and curation, manuscript preparation, review and editing. Department of Orthodontics and Dentofacial Orthopedics, Medical Faculty, School of Dental Medicine, University of Bern, Bern, Switzerland.; b Martina Ferrillo PhDs, Department of Health Sciences, University of Catanzaro ‘Magna Graecia’, Catanzaro, Italy. Manuscript review and editing.; c Nikolaos Pandi Associate Professor, Department of Orthodontics and Dentofacial Orthopedics, Medical Faculty, School of Dental Medicine, University of Bern, Bern, Switzerland. Statistics, validation, and manuscript review.; d Dimitrios Kloukos Senior Lecturer, Department of Orthodontics and Dentofacial Orthopedics, Medical Faculty, School of Dental Medicine, University of Bern, Bern, Switzerland; Department of Orthodontics and Dentofacial Orthopedics, 251 Hellenic Air Force & VA General Hospital, Athens, Greece. Conceptualization, methodology, and original draft preparation.; e Padhraig S. Fleming Professor, Dublin Dental University Hospital, Trinity College Dublin, Ireland. Supervision, manuscript review and editing.; f Christos Katsaros Professor, Department of Orthodontics and Dentofacial Orthopedics, Medical Faculty, School of Dental Medicine, University of Bern, Bern, Switzerland. Conceptualization, methodology, manuscript review and editing.

**Keywords:** gingival recession, periodontal disease, orthodontic retainers, orthodontics

## Abstract

**Purpose:**

The prevalence of gingival recession in orthodontically treated patients and the relative impact of retainer type on its occurrence remain poorly understood. The objective of this study was to investigate the association between previous orthodontic treatment and retainer type on the long-term prevalence of gingival recession and to evaluate the role of other patient-related factors, such as gender, age, smoking and gingival phenotype.

**Materials and Methods:**

We included subjects both with and without a history of previous orthodontics (at least 5 years post-treatment). The periodontal status assessment and the presence of gingival recession were recorded. A generalised estimating equation (GEE) logistic regression model was used to examine the effect of the mode of retention and tooth type on recession adjusted for age, smoking, gender and gingival phenotype.

**Results:**

A total of 251 individuals (mean age of 32 ± 9.43 years) were included. Ninety-nine (39.4%) had a history of orthodontics with an observation period of 15.7 years. Those undergoing orthodontics followed by fixed retention had the highest prevalence and magnitude of recession; a history of orthodontics was statistically associated with the occurrence of recession (odds ratio: 2.40; 95% CI: 1.52; 3.82; P < 0.001). Both age and the presence of a thin gingival phenotype were significant predictors for recession (P < 0.001). The adjusted probabilities of recession per tooth indicated that the mandibular central incisors had the highest probability for recession, with either a fixed or removable retainer.

**Conclusions:**

Based on this observational study, the provision of orthodontic treatment followed by removable or fixed retention had a bearing on the occurrence of recession. The aetiology of gingival recession is multifactorial with a thin periodontal phenotype, age and smoking history being risk factors, while mandibular central incisors are particularly susceptible.

Orthodontic relapse can be defined as a partial or complete return of the teeth to the unfavourable pre-treatment position.^2^ Relapse is thought to be unpredictable having a complex and multifactorial aetiology.^29^ There are two major contributors to post-treatment change including both true relapse and maturational effects.^29^ As such, only 10% of orthodontic patients retain acceptable mandibular arch alignment 20 years post-retention.^16^ The near pervasive and unpredictable nature of post-treatment change dictates a reliance on a conservative, near-universal and indefinite retention.^7,25^

Both removable and fixed retainers are, therefore, routinely recommended to preserve long-term stability and avoid post-treatment occlusal change.^3,19^ While removable retainers allow easier maintenance of oral hygiene, their performance depends on patient compliance.^32^ Conversely, fixed retainers are less contingent on patient compliance and have been shown to be more effective in maintaining the alignment of the anterior teeth in the medium to long term.^6,19^ However, fixed retainers are prone to failure and susceptible to plaque accumulation, which may culminate in periodontal inflammation.^6,17^

It is also postulated that fixed retention might be considered a risk factor for the development of gingival recession,^15,26^ although studies are conflicted in this respect.^4,8,10^ Indeed, while the mandibular central incisors are more prone to the development of gingival recession, the relative contribution of orthodontic mechanics, the movement of teeth out of the alveolar envelope during the active phase,^9^ and the presence of fixed retainers in the development of gingival recessions is uncertain.^31^ Notwithstanding this, it is accepted that unplanned tooth movement introduced by active or deformed fixed retainers may be accompanied by adverse periodontal effects.^12,23^

Understanding tissue reactions during orthodontic or post-orthodontic movements is essential for clinicians when devising a comprehensive orthodontic-periodontal treatment plan.^1^ Moreover, the oral microbiota may play an important role in the overall health and symbiosis status of the individual. Deviations from the state of symbiosis lead to dysbiosis and an increased risk of pathogenicity. Deviations can occur not only from daily life activities but also from orthodontic interventions.^24^

The prevalence of gingival recession in previously treated groups and the relative impact of retainer type on the occurrence of recession remains poorly understood.

Therefore, the aim of the current study was to investigate the association between previous orthodontic treatment and retainer type and the presence of gingival recession in the long term and to evaluate the role of other patient-related factors, such as gender, age, smoking, and gingival phenotype.

## MATERIALS AND METHODS

Ethical approval for this retrospective study was obtained from the Ethical Committee of 251 Hellenic Air Force General Hospital, Athens, Greece. All participants were asked to read and sign an informed consent form. The study was undertaken in accordance with the Declaration of Helsinki of 1975. The study was performed in accordance with the STrengthening the Reporting of OBservational studies in Epidemiology (STROBE) Guidelines.^30^

### Participants

We included volunteers from both the Periodontology and Orthodontics Departments of 251 Hellenic Air Force General Hospital. Subjects with or without a history of previous orthodontic treatment were selected. Previous orthodontic treatment had to include fixed appliances (brackets in both jaws) or removable aligners in both jaws, with pre-existing removable appliances (first treatment phase) not being mandatory as an inclusion criterion. This implies that included participants had by all means been treated by fixed appliances or aligners, but not always by a first phase with removable ones (before brackets or aligners). Extraction cases were also accepted.

We excluded the following subjects: (1) history of diabetes; (2) pregnant or breast-feeding women; (3) current use of antibiotics or the need for antibiotic prophylaxis for periodontal assessment; (4) diagnosis of periodontal disease before or after orthodontic treatment; (5) professional dental cleaning within the last 4 months; or (6) intake of medication with any known effect on the periodontal soft tissues.

Those reporting a history of orthodontic treatment were included if they were at least 5 years into retention with a fixed bonded or removable retainer. A range of approaches to retention were considered including fixed and removable retainers or a combination of these.

### Periodontal Assessment

A trained periodontist at the Periodontal Department of 251 Hellenic Air Force General Hospital performed the periodontal assessment with periodontal status assessed according to accepted criteria.^30^ Specifically, a score between 0 and 3 was assigned for each patient overall as follows: 0: healthy periodontium; 1: healthy treated periodontium; 2: gingivitis; 3: periodontitis. The presence of gingival recession (REC) was also recorded being defined as the distance between the cemento-enamel junction (CEJ) and the free gingival margin. These measurements were recorded for the labial surfaces of all teeth. The surfaces of each tooth were divided into thirds to demarcate mesial, middle and distal surfaces, using vertical lines based on the position and morphology of the dental papilla. All measurements were obtained by the same researcher using a periodontal probe (NC 15, Hu-Friedy) and were recorded in millimetres. A standard single-ended, colour-coded periodontal probe was inserted 1 mm into the gingival sulcus under natural light without magnification. The gingival phenotype was classified as a binary variable by assessing the visibility of the periodontal probe through the gingiva.^5,14^

### Statistical Analysis

Demographic and clinical data were calculated with conventional descriptive statistics. Continuous variables were expressed as means and standard deviations, while the categorical variables were expressed as absolute numbers and percentages. A generalised estimating equation (GEE) logistic regression model was fitted with robust standard errors to examine the effect of the mode of retention and tooth type on recession adjusted for age, gender and gingival phenotype. A P-value of <0.05 was considered statistically significant. All statistical analyses were conducted with STATA® version 17 software (Stata Corporation, College Station, TX, USA).

## RESULTS

A total of 251 individuals with a mean age of 32 ± 9.43 years met the eligibility criteria and were included in the final analysis. Most included participants were male (n = 216; 86.1%). Ninety-nine participants (39.4%) had a history of orthodontics, with the mean period between the commencement of orthodontics and follow-up being 15.69 years. Four patients were extraction cases (either first or second premolars). 98 patients received retention after their orthodontic treatment; out of them, 41 (41.42%) received removable retainers, and 57 (57.58%) received a fixed retainer bonded on all six anterior teeth. From the group with fixed retainers, 31 out of the 57 received a twisted wire fixed retainer (31.32%), again bonded on all anterior teeth. One participant did not receive any form of retention after orthodontics. Gingivitis was identified in 98 (39.04%) and periodontal disease in 13 (5.18%). A thin periodontal phenotype was noted in 42 participants (16.73%; Table 1).

**Table 1:** Demographic and clinical characteristics of the sample (n = 251)

Age (years)	32.00 ± 9.43
Gender	(n, %)
Male	216 (86.06)
Female	35 (13.94)
Smokers (n,%)	57 (22.71)
Previous orthodontic treatment	(n, %)
Fixed	95 (37.85)
Removable	4 (1.59)
Retention type	(n, %)
Removable	41 (41.41)
Fixed	26 (26.26)
Twisted fixed	31 (31.32)
No retention	1 (1.01)
Periodontal status	(n, %)
Healthy periodontium	134 (53.39)
Healthy treated periodontium	6 (2.39)
Gingivitis	98 (39.04)
Periodontitis	13 (5.18)
Gingival phenotype	(n, %)
Medium/thick	209 (83.27)
Thin	42 (16.73)
Toothbrush type	(n, %)
Soft/medium	228 (90.84)
Hard	23 (9.16)
*Continuous variables are expressed as means ± standard deviations; categorical variables are expressed as counts/percentages.

Fifty-one of the untreated subjects (33.55%) had recession defects, with just three of these having a single defect, 11 having two or three defects and 37 subjects with more than three recession defects. In the treated sample with either removable or fixed retainers, 67 subjects (67.68%) had recession defects overall. Fifty of them presented with more than three recession defects (50.51%) (Table 2).

**Table 2:** Occurrence of single or multiple recessions in all teeth for the overall sample and based on previous orthodontic treatment and retention

	No orthodontics (n = 152)	Previous orthodontics (n = 99) followed by removable retainer (n = 41) or fixed retainer (n = 57) or no retention (n = 1)
n (%)	n (%)
Participants with one recession	3 (1.97)	5 (5.05)
Participants with two recessions	7 (4.60)	7 (7.07)
Participants with three recessions	4 (2.63)	5 (5.05)
Participants with >3 recessions	37 (24.34)	50 (50.51)
Participants with recessions	51 (33.55)	67 (67.68)
*Categorical variables are expressed as counts/percentages.

**Fig 1:**
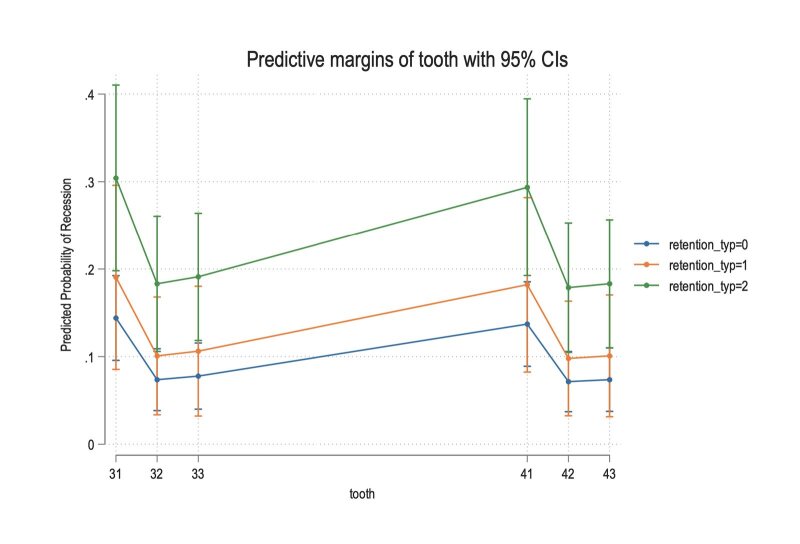
Adjusted probabilities of recession per tooth for the different retention methods. Teeth 31 and 41 have the highest probability for recession (approx. 0.3 with fixed retention). *Abbreviations: retention_typ = 0 : untreated subjects; retention_typ = 1: removable retention; retention_typ = 2: fixed retention.*

The adjusted probabilities of recession per tooth for the different retention methods are displayed in Figure 1 for the mandibular anterior teeth. Specifically, the mandibular central incisors had the highest probability for recession, which was more for teeth with fixed retention than for those with removable retention.

In terms of the severity of gingival recession at the mandibular front teeth, the untreated group experienced fewer recessions overall. The group of subjects who underwent orthodontics followed by fixed retainer showed a higher amount of recession defects. Specifically, 14.04% of those having fixed retention had recessions on two teeth, 5.26% on three teeth and 10.53% had recessions on all four anterior teeth. The corresponding figures in the subset (n = 153) who did not have orthodontics were 7.24%, 0.66% and 6.58%, respectively (Table 3).

**Table 3:** Severity of recession at the mandibular anterior incisors

Participants with:	No previous orthodontics (n = 152)	Previous orthodontics (n = 98)*	Orthodontics followed by removable retainer only (n = 41)	Orthodontics followed by fixed retainer(n = 57)
n (%)	n (%)	n (%)	n (%)
No tooth with recession	129 (84.87)	66 (67.34)	31 (75.61)	35 (61.40)
One tooth with recession	1 (0.66)	9 (9.09)	4 (9.76)	5 (8.77)
Two teeth with recession	11 (7.24)	11 (11.11)	3 (7.32)	8 (14.04)
Three teeth with recession	1 (0.66)	3 (3.03)	0 (0.00)	3 (5.26)
Four teeth with recession	10 (6.58)	9 (9.09)	3 (7.32)	6 (10.53)
*Excluding the one participant who received orthodontic treatment but no retention.

Based on the adjusted GEE regression model, a history of orthodontics (odds ratio: 2.40; 95% CI: 1.52; 3.82; P < 0.001) had a bearing on the occurrence of recession (Table 4).

**Table 4:** Generalised estimating equation (GEE) logistic regression analysis on the association between the occurrence of recession at all teeth and previous orthodontic treatment adjusted for smoking, age, gender and gingival phenotype

Covariate	Odds Ratio	95% Confidence interval	P>|z|
Previous Orthodontic treatment
No previous treatment	1		
Previous treatment	2.40	(1.52 to 3.82)	<0.001
Smoking			
No*	1		
Yes	1.69	(1.03 to 2.76)	0.038
Age			
Per unit	1.07	(1.04 to 1.10)	<0.001
Gender			
Male*	1		
Female	0.88	(0.49 to 1.58)	0.667
Gingival phenotype			
Medium/thick *	1		
Thin	3.31	(1.94 to 5.64)	<0.001
* Baseline category

However, in the adjusted GEE regression model solely for the mandibular incisors, a history of orthodontics followed either by removable (odds ratio: 1.65; 95% CI: 0.70; 3.88; P = 0.251) or fixed retention (odds ratio: 1.90; 95% CI: 0.65; 5.52; P = 0.238) did not reveal a bearing effect on the occurrence of recession, (Table 5).

**Table 5:** Generalised estimating equation (GEE) logistic regression analysis on the association between the occurrence of recession at the mandibular anterior teeth (central and lateral incisors) and retention modality adjusted for smoking status, age, gender and gingival phenotype

Covariate	Odds ratio	95% Confidence interval	P>|z|
Retention type			
No retention*	1		
Removable retention	1.65	(0.70 to 3.88)	0.251
Fixed retention	1.90	(0.65 to 5.52)	0.238
Smoking
No*	1		
Yes	2.54	(1.31 to 4.95)	0.006
Age			
Per unit	1.07	(1.03 to 1.11)	0.001
Gender			
Male*	1		
Female	1.23	(0.41 to 3.67)	0.712
Gingival phenotype			
Medium/thick *	1		
Thin	6.76	(2.24 to 20.36)	0.001
* Baseline category

## DISCUSSION

Based on the present findings, the provision of orthodontic treatment followed by removable or fixed retention had a significant bearing on the occurrence of recession with other factors, including the presence of a thin periodontal phenotype, being more influential in this respect.

Clinical studies so far have shown that the proclination of teeth and movement of the incisors out of the osseous envelope of the alveolar process may be associated with a higher tendency for developing gingival recessions.^9^ The amount of recession, nevertheless, found in studies with statistically significant differences between proclined and not proclined incisors is small, and the clinical consequence is questionable.^9^ Moreover, the putative link between orthodontic retention and recession is largely based on case series and isolated case reports of extreme complications.^12,15,23^ Notwithstanding this, the impact of both removable and fixed retainers on long-term periodontal health is not fully understood.

In the context of the previous studies, Khalil et al^13^ failed to detect an association between fixed retention and mandibular anterior recession when compared to untreated controls. The authors reported a mean recession of 0.1 ± 0.2 mm in both groups at the 10-year follow-up. We did not measure recession as a continuous variable instead classifying recession as a binary variable as this better reflects the associated requirement for intervention. Similarly Juloski et al,^10^ in a 5-year follow-up of mandibular lingual retainers, highlighted that the occurrence of recession was not influenced by orthodontic treatment, the presence of a retainer, age or gender. However, they did also observe a predilection for recession on the central incisors particularly in those with fixed retention.^10^ Conversely, Levin et al^15^ observed a higher prevalence of gingival recession among those with fixed retainers compared to post-orthodontic patients without fixed retainers and untreated subjects approx. 5 years post-treatment. The authors also linked more gingival positioning of fixed retainers both to recession and local inflammation. It is conceivable that the longer period of follow-up in the present study may contribute to a dilution of any shorter-term effect associated with the presence of the fixed retainer.

The conflicting findings concerning the possible link between orthodontic treatment, retention and recession may relate to the complex aetiology of gingival recession, with orthodontic treatment and retention representing two environmental factors.^21^ Indeed, periodontal health is inextricably linked to plaque accumulation and oral hygiene status. Moreover, a very low quality of evidence concerning the effects of different retainers on periodontal outcomes and the related effect concerning calculus formation has been highlighted.^31^ It is also accepted that mandibular incisal proclination of approx. 8 degrees is associated with a 50% risk of inducing 2 mm in loss of alveolar bone height with movement of the incisors out of the osseous envelope predisposing to the development of gingival recession at some point.^9,18^ It is also noteworthy that fixed retention may be prescribed in those in whom incisal advancement has arisen; as such, isolating the effect of the retainer from the impact of the orthodontic tooth movement is complicated. Equally, the compatibility of mandibular fixed retainers with periodontal health and marginal bone levels has been shown both in the short and long term.^4,31^ As such, the development of recession cannot be ascribed exclusively to orthodontic treatment or retention, with these interventions being interlinked and likely subordinating to the effects of the periodontal phenotype and other patient factors.

In keeping with previous studies,^11,26–28^ tooth type was a significant predictor for recession, with the mandibular central incisors appearing to be particularly prone to recession. Renkema et al^26^ recorded the most defects on central incisors, followed by canines and mandibular lateral incisors 5 years post-treatment, mirroring our findings. The presence of a thin periodontal phenotype was a significant predictor for recession, underscoring the importance of evaluating periodontal phenotype during orthodontic assessment with a thin-scalloped phenotype regarded as at higher risk of recessions compared to thick-flat phenotype.^22^

In terms of limitations, this was a cross-sectional study and therefore may have been susceptible to selection bias^20^; additionally, gingival recessions before orthodontic treatment have not been recorded and other confounding factors may not have been adequately controlled as, for example, home oral hygiene practices. These are inevitably drawbacks in non-prospective studies. Moreover, a wide age range and a larger proportion of male participants were included in this research; this has to be taken into consideration when appraising the results and could attributed to the specific study setting. Finally, the role of extractions could also not be investigated due to the small sample of patients treated with extraction protocol. Nevertheless, an overall large sample allowing for both evaluation of the effects of fixed and removable retention, and indeed permitting particularly prolonged evaluation, was possible.

## CONCLUSIONS

Based on the current results, orthodontic treatment followed by removable or fixed retention appeared to be significantly associated with recession. The aetiology of gingival recession is multifactorial with a thin periodontal phenotype, age and smoking history being. Mandibular central incisors appear to be the most susceptible teeth, especially in the presence of a fixed retainer. Further prospective controlled evaluation of the contributors to the development of gingival recession would be welcome.
